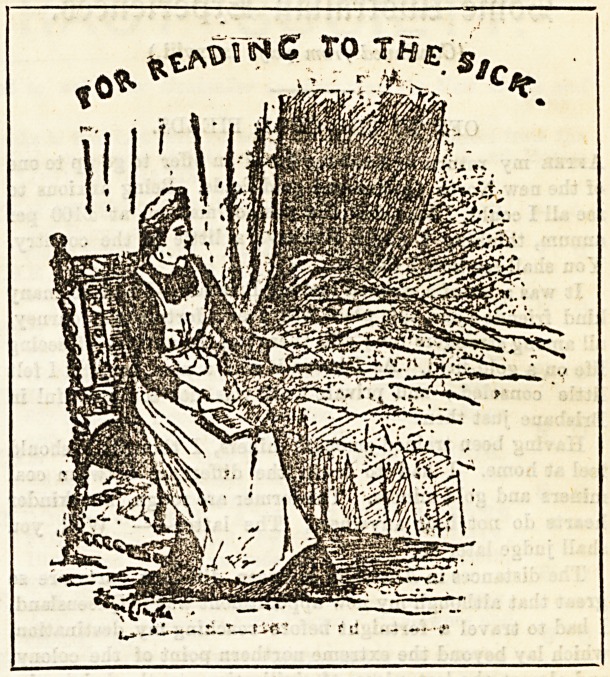# The Hospital Nursing Supplement

**Published:** 1892-12-17

**Authors:** 


### "The Hospital" Nursing Mirror.

Being the Extra Nursing Supplement of "The Hospital" Newspaper.

[Contributions for this Supplement should be addressed to the Editor, The Hospital, 140, Strand, London, W.C., and should have the word "Nursing" plainly written in left-hand top corner of the envelope.]

### En Passant.

LADY DUFFERIN'S HOSPITAL, AGRA.—Miss Lilian Trewby, L.R.C.S., L.R.C.P.Eng., who has been in private practice in this country as a lady doctor, left England on Thursday for India, having been appointed senior house surgeon of Lady Dufferin's Hospital at Agra. Miss Trewby was educated at the London School of Medicine for Women.

LYME REGIS.—The Committee of the Cottage Hospital, Lyme Regis, Dorset, have decided that whenever a vacancy of a bed occurs they will admit convalescent patients who need the finishing touch of health so often required ere they are ready to begin work again in sober earnest. Any-one admitted must be able to give a reference as to good character, and the charge will be ten and sixpence a week. In this lovely little place on the sea coast such an arrangement will prove most desirable.

A NEW NURSING SCHOOL.—Many readers of The Hospital will be interested to hear of the new nursing school which is shortly to be opened at Mill Road Infirmary, Liverpool. There is a fine new nursing home, and the rules for probationers are on the lines of those already existing in the best general hospitals. We hear that the Matron has received an immense number of applications from would-be probationers, but she has still a few vacancies for duly qualified charge nurses.

AMERICAN NOTES.—A proposal is a foot in New York to start a Nurses' Association and Club House where nurses can reside, and where all the advantages of a good club and library will be obtainable.—The Training School at the John Hopkins Hospital, Baltimore, is taking a certain number of graduates from other schools for the course of invalid cookery instruction.—The English letters now appearing in the *Trained Nurse* are evidently written by a "trained nurse," and fairly reflect the views and progress of the English nursing world.

"LONG EXPECTED."—It is with real pleasure that we find ourselves able to tell our readers that training in the care of mental cases is at last within a nurse's reach. It is, thanks to Dr. Greene and Dr. Harding, and the Berrywood Asylum, Northampton, authorities that this much-needed scheme has definitely developed. The following are some of the conditions on which probationary nurses will be received at Berrywood: A fee of ten guineas will be payable on entering; the probationers will, after passing an examination, practical and theoretical, receive a certificate of competency to undertake the nursing of mental cases. Should the probationer leave at the expiration of six months, she may receive a letter stating she has had six months' residence as a probationer; but the full certificate can only be granted at the expiration of the full period of twelve months. These are the most important points. Nurses will, of course, have to conform to all the usual rules, and will wear uniform which, with board, lodging, and washing, will be provided for them. How welcome this news will be to many nurses we well know. The general public has no conception of the difficulty there often is in finding a really suitable nurse for a mental case, but now that difficulty bids fair to be soon overcome. Sincere acknowledgments are due to the authorities of Berrywood for the public spirit they have displayed in the matter.

THE SCILLY ISLES.—A trained nurse arrived at the Isles of Scilly on Saturday, the 3rd inst., to do duty under the Dorrien-Smith Nursing Association, started by voluntary contributions as a memorial to the late Mrs. Dorrien-Smith. Another nurse is expected shortly.

A PROVIDENT NURSING SCHEME.—The sum of £8,300 is left, under the will of the late Mr. Sands-Cox, for the formation of a provident nursing scheme which is to benefit Nechells. The scheme is to be entirely a provident one and will be devoted to the aid of subscribers only, but the annual subscription will, it is hoped, not exceed two shillings, so that it will be practically within the reach of everybody.

SECTARIANISM.—Mrs. Blackmore, the Stamford town nurse, has received notice to leave on account of her having entered the Roman Catholic Church. The editor of *Truth* takes the matter up warmly and says: "Such an exhibition of sectarian bigotry and sour intolerance seems incredible in these days, and the matter is to be brought under the notice of the managers of the Central Nursing Association. The institution at Stamford is supported by persons of all creeds, and the work of a nurse has nothing whatever to do with religion." Has any case ever occurred where a nurse has attempted to proselytize her patients?

FEES AND CO-OPERATION.—The revolution as regards nurses' fees has certainly worked good on all sides, and although the number of self-supporting co-operations are as yet few in number, the practice of granting fair percentages on nurses' earnings is fast gaining ground amongst private institutions. The last we hear of is at Woodland House, Matlock Bath, started by Miss Mary Ferry, who takes 5s. in the guinea, and who undertakes to nurse any nurse who may be taken ill, without extra cost, and whose rules seem very just and reasonable. We should like to hear of more nurses banding together, and it is with great pleasure that we see that the Nurses' Co-operation for Glasgow and the West of Scotland is really in a fair way to realisation. These co-operations work good in two ways, they not only obtain the wages for the nurses themselves, but they will in time do a great deal to keep up the standard of efficiency, for the reason that to retain an indifferent nurse means failure to the entire community.

A TRIBUTE.—At the last meeting of the Council of the Royal National Pension Fund for Nurses, Mr. Walter H. Burns in the chair, the death of Dr. J. C. Steele, of Guy's Hospital, having been reported, it was moved by the Chairman seconded by Mr. Burdett, and resolved: "That the Council of the Royal National Pension Fund for Nurses have heard with deep regret and sorrow of the lamented death of their colleague, Dr. Steele, who had been a member of the Council since the institution of the Fund. Dr. Steele always displayed the keenest interest in the progress of the Fund, and was ever ready to do his utmost to promote the welfare of nurses by heartily co-operating to alleviate the sorrows and to minimise the risks attaching to the calling of a nurse. The Council desire to tender to Mrs. Steele and the family the expression of their sympathy with them in this heavy trial, and to place on record their appreciation of the services which Dr. Steele rendered not only to the Royal National Pension Fund for Nurses, but to the progress of nursing generally."

### The Development of Children by Gymnastics.

### I.—POPULAR NOTIONS.

The opinion that gymnastic exercises should form a regular part of the education of girls as well as of boys has certainly of late years become more general among European peoples, though it is far from holding the prominent place it merits. With a view to establishing it on a higher level, it is proposed to publish a few articles on the various methods popular in this country.

There is still a lingering feeling amongst a certain class of parents and guardians—possibly the remnant of the "papa, potatoes, prunes, and prism" system—that it is waste of time to take the children away from their books to go through a series of exercises which seem "almost like games," but it is very evident that such have not had actual experience in teaching, or certainly not in contrasting classes of girls or boys brought up under the uninterrupted "cramming" system and those who have bodily recreation—for, in the primary sense of the word, drill *is* "re-creation"—interspersed with headwork.

It is only natural that a continued *strain* on any part of the human system should tend to deaden its power of action, especially before it is fully developed, as in the case of children; and though one may change the course of study from languages to history, history to mathematics, mathematics to science, and so on, yet the one is but a greater effort of brain-work than the other. Now, by the introduction of gymnastics into the daily routine, the body, necessarily cramped by continuing in more or less the same attitude throughout the classes, is invigorated and rested by change of position, and the contracted muscles, being relaxed and brought into play, are enabled to impart new energy to the whole body and indirectly to the mind, since the power to concentrate one's attention is always greatest when the irritation of physical worries is removed.

It would be found, in the long run, to be economy, of both time and trouble, to curtail the lessons in favour of gymnastics. A half hour's work done brightly and intelligently is worth weeks of study—if we may so misuse the word—done in a sleepy, half-hearted spirit produced by lack of energy, a condition which might easily be avoided by a few vigorous exercises.

Surely if this were taken into account we should have all "our daughters" joining the drill class or the local gymnasiums, which are now only few and far between, considering the vast population, a very different state of affairs to that to be met with even in times so remote that they border on the pre-historic, when the Olympic Games stood first in the opinion of the ancient world, and the gymnasium was one of the chief features in every city, the resort of high and low alike. Looking to the Continent, we find His Imperial Majesty standing forth as the patron of all athletic games; across the Atlantic we have the question of physical training making rapid strides; why should England be the last to cultivate in her girls a healthy, vigorous, and graceful development of nature's free gifts?

Since gymnastic exercises have become a part of the regular army "drill," the soldier is far more agile, his muscles more developed, and his strength greater than would be the case if this branch were wanting; but if each unit in the force had undergone this training from childhood, our army, as a whole, would present a front far more formidable and imposing than it does even now. The unmistakable advantages gained by this special training, even when taken up by men of advanced years, leads us to suppose that a far better result would have been attained had they begun in childhood, and that by easy and gradual stages they might have been brought up to a standard far higher than the present, but one which we hope will be general before many years are gone.

In his speech during the last annual meeting of the Drexel Institute at Philadelphia, M. Demeny called attention to an important fact, namely, that, as art is necessary to the cultivation of flowers, so is physical culture essential to the complete development of the body. The human frame is not necessarily perfect; indeed, the tendency in this world is rather to deterioration than to perfection; from childhood onwards, strength, symmetry, health can only be gained, and when gained, maintained, by the most untiring vigilance. In the ancient Spartan days delicate infants were considered as unworthy to live, and were abandoned on Mount Tagetus to die; now the weak are guarded and cherished most zealously and, by artificial means, we strive to give them what Nature seems loath to supply. Thus it is that we want to assure all who are anxious to create in their children a fund of health whereon to draw throughout life, that weak spines, delicate chests, and other infirmities which manifest themselves at the critical stages of "growing," generally vanish, or at least, diminish after a course of judicious daily calisthenics. I emphasise "judicious," for many people disregard the truth that "one may have too much of a good thing," and, in consequence, they strain the weak part rather than strengthen it gradually by carefully-chosen exercises.

We strive to do our utmost for the mental training of the young; why should we neglect their physical development? When we see a tree with its branches on the one side fully grown, while on the other they are stunted and withered, we know at once that a course of ungenial influences has caused the discrepancy. The one-sided perfection contracts only the more painfully with the adjoining imperfection; the whole is an unsightly and distressing picture which by care and cultivation might have been averted. Surely the cultivated mind is worthy of something better than a deformed or an undeveloped body.

Like the Athenians of old, we strive after all things that are new: we hail with outstretched arms the latest inventions or discoveries in medicine, but ignore the fact that we are neglecting the most natural cure for weakness, namely, bodily exercise, which remedy is open to all. Like Naaman, we want some "great thing" proscribed for us, and in our pride and ignorance spurn the seemingly unimportant "washing in Jordan."

Besides the actual bodily health we have to take into consideration the training of the "will." We have all realised at times what annoyance can be felt in watching a dilatory child; it seems as if there were a "missing link" between the mental command and the physical fulfilment of it, a link only to be supplied by "culture" of will. Each member of the body should be subservient to the will; consequently, a thoroughly healthy state can only be enjoyed when all parts work together in harmony, yet when every action takes place with such promptitude that each limb would seem to have a special will-power of its own. The happy combination of mental, physical, and will-power gives vivacity to action, elasticity to the step with a general easiness, and grace of movement, a goal to which all will acknowledge that it is worth pressing.

Of course, no fond parent will acknowledge that his or her child in particular is what the world calls "spoilt"; but such children undoubtedly *do* exist, and on them a brief attendance at a "drill" class has the best possible influence in the formation of character. Home "commands" in such cases are apt to be dallied with and even ignored altogether; but the sharp orders, "Right," "Left," "Advance," "Charge!" brook no half-hearted obedience, and in carrying them out the child is unconsciously trained to look on the commands of those in authority as something more than mere words, and his own "will" as a power given, not exclusively for his sole pleasure, but meant also to bend to superior wisdom and experience. If this could be instilled into the very "being" while young, we might reduce the overwhelming amount of selfishness which is increasing daily around us.

### The Royal British Nurses' Soiree.

Between seven and eight hundred people gathered at the fifth annual conversazione of this Association, which was held on the evening of Wednesday, December 7th, at the Royal Institute of Painters in Water Colours at Prince's Hall. The Institute is a delightful place for a gathering, for besides being picturesque, the many entrances and galleries prevent any sort of crushing, that bugbear of "At Homes" and large social functions generally.

The reception of nurses commenced at half-past eight, and Miss Isla Stuart, the Matron of St. Bartholomew's, Mr. Pickering Pick, and Sir J. Crichton Browne stood at the central door to receive them. Matrons, sisters, and nurses, all dressed simply in their becoming uniforms, soon made the rooms an animated scene.

The Bijou Band played delightfully, and as we sat listening to the music chatting with our friends or looking at the pictures the time flew very fast, and at about ten o'clock the band struck up "God save the Queen," and Her Royal Highness the Princess Christian, President of the Association, arrived. The Princess was accompanied by Prince Christian, her daughter, and the Baroness von Egloffstein, and as soon as she had mounted the platform the presentation of badges took place. As far as we could see there were twenty-six recipients of the badge, and afterwards several matrons and sisters were presented to her Royal Highness. The Royal President wore a very handsome dress of brocade, with sleeves of old-rose colour, and she carried a beautiful posy, which was presented to her by Miss Hogg, Chief Sister at Haslar.

We noticed among the many present Lady Jeune, Sir Richard Webster, Lord Halsbury, Mr. John Morgan, Mr. Brudenell Carter, Dr. Begley Thorne, Mr. and Mrs. Langton, Mr. Owen Lankester, Miss Lankester, Miss Butler, of the Samaritan Hospital, Miss Ridley, of the Regent's Park Hospital for Paralysis, Mrs. Macintyre, the Matron of the Home of Rest at Brighton; Dr. and Mrs. Bedford Fenwick, (the latter in uniform); Miss Cooper, of the Victoria Hospital; Miss Hughes, the Matron of the Kensington Infirmary; Miss East, from the National Hospital, Queen Square; Miss Beachcroft, from the Lincoln County Hospital; Miss Mackay, from the Golden Square Hospital; Miss De Pledge, and Miss Thomas, who has so successfully started the Domestic Technical School in Oakley Street, Chelsea. Miss Robins, the new Secretary of the Royal British Nurses' Association, was very energetic, and was besieged on all sides by members of the Press, who wanted to know anything and everything about everybody. Miss Robins was Assistant Secretary to the National Health Society, and helped in a great measure the starting of the Society's technical County Council lectures. We did not see Miss Catherine J. Wood, and we were told she was not present. At half-past ten Mr. Corney Grain was seen coming in, and immediately there was a rush from all sides to get near the piano. He certainly had a very appreciative audience, and sang song after song much to everybody's enjoyment. Altogether the evening was unanimously voted a very pleasant one.

### Notes and Queries.

### Queries.

(34) *Midwifery at Birmingham.*—Will some certificated midwife tell where one can learn midwifery in or near Birmingham without entering a hospital?—*Nurse Charlotte.*

(35) *The Latest Hospital Authority.*—"Can you tell me what experience of hospitals Mr. H. S. Alexander has had? He is the latest critic, but his charges seem to have so familiar a ring as to appear stale from repetition."—*Curious.*

(36) *Berm ndsey.*—Can you tell me where I can procure "The History of Guy's Hospital," noticed in The Hospital.

### Answers.

(35) *The Latest Hospital Authority* (*Curious*).—We learn on inquiry that the gentleman mentioned has recently attained his majority, and has qualified as a hospital critic by a few weeks' residence as a patient at Gordon House.

(36) *Bermondsey.*—"The History of Guy's Hospital" is published by Ward, Lock, and Bowden Warwick House, Salisbury Square, price 10s. 6d.

### Working and Waiting.

"The following is the form which all nurses who volunteer for cholera nursing are required to sign: 'I, the undersigned, hereby request that my name may be placed on the roll of volunteer nurses for cholera, and in the event and in consideration of my request being complied with, I promise to undertake the nursing of cholera patients in whatever part of the United Kingdom the Special Committe for Cholera Nursing of the Royal British Nurses' Association may decide that my services are required; and I agree to be bound by the rules of any institution, and to act under the orders of any sanitary authority or the authorised medical officer of any sanitary authority, or any registered medical practitioner to whom I may be delegated.'"

This was the form as it appeared in the *Daily News* last month, and it is certainly a very plain and practical statement, and one which can leave no doubt on the mind of any reader as to the pledge required from nurses enrolling themselves as cholera volunteers. H.R.H. Princess Christian shows in this, as in many of her other undertakings, an undoubted interest in the well-being of the community. She certainly has lent her name to a plan which will ensure an adequate supply of attendants for those who may suffer from cholera in the future. Of course, we imagine that some fund is already in existence which will provide for the maintenance of the enrolled nurses, who are invited to hold themselves in readiness for "the nursing of cholera patients in whatever part of the United Kingdom" their services are demanded. However willing and enthusiastic women may be, only a small proportion of the sex are endowed with sufficient private means to justify them in throwing up private nursing or hospital permanent appointments for the uncertain prospects of remunerated or unremunerated cholera work. No conscientious probationers or trained nurses can pledge themselves to desert their posts for any summons, save and except those of their own institutions, and naturally the latter are all willing to second the energetic movement of Her Royal Highness. When cholera appears in England we can rely implicitly on our countrywomen doing their duty honourably and honestly, and the sanitary officers will find themselves ably supported by the heads of hospitals and all other nursing institutions as need arises. If, however, any previous enrolment of individuals is considered desirable it would certainly be well to publish, for the information of nurses and the public generally, some details as to the fund which provides board, lodging, and laundry for a body of women who are philanthropic enough to give up present certainty for vague, if romantic, future employment.

It is well to remember that it is not only the possible epidemic which we have to provide for. There are the ordinary every-day sick people to be tended still. There are invalids in wards and in private houses each hour of the day, and each day of the week. Scarlatina is still with us, and diphtheria is not stamped out; measles and typhoid, pneumonia, brain disease, and serious operations claim the trained nurse's care from day to day. Will any employers or any wise doctors consent to engage persons who tell them that they have a second string to their bow in the shape of cholera nursing? We fear not, and we are, of course, safe in asserting that no worthy woman could apply for present work without stating that she has pledged herself to go elsewhere. A trained nurse's reputation is, in effect, her livelihood, and she has to guard it from any shadow of dishonourable double dealing.

Whatever views the nation may hold as to the probable visitation of cholera next year, it is not likely to wish nurses to bear the financial responsibility of the crisis. We can trust the fairness of doctors and officers of health, and we know that most of them will give due warning to all nursing schools of any prospect of an unusual drain upon their resources. Thus medicine will find its handmaid, "skilled nursing," following where it leads, and present duties in nowise neglected for future possibilities.

### Nurses in Germany.

Surely English women should feel a special interest in the progress made by the nursing profession in Germany, for certainly the advancement is, in part, at any rate, due to the efforts and influence of our own Princess Royal, better known at the present day as the Empress Frederick.

The parish or district nurses (deaconesses) are universally employed. They belong to one of the religious orders and are provided with rooms, food, and clothing, the latter including not only uniform, but every article of apparel down to their serviceable boots. No salary beyond a trifling sum as "pocket-money" is given to or demanded by these worthy women who have been trained in one of the recognized hospitals.

It is generally arranged for two or more of the district nurses to live together, a general sitting-room and separate bedrooms being provided.

With regard to the training of nurses there are certain regulations made by Government to be observed, and the granting of certificates is similarly supervised.

Private nursing is still in its infancy in Germany, and no single nurse could make a living "on her own account" there as she does here. The religious orders, male and female, and of various denominations, supply most of the nurses required by the rich and poor. The great middle-class, to a great extent, look after each other whenever possible. This cannot be always as satisfactory to the doctor as the presence of a trained and experienced nurse in any grave or difficult case of illness. Doctors themselves appear to receive very low fees in Germany, and therefore they pay more frequent visits, in cases of ordinary sickness, and do most "dressings" themselves. Permanent military hospitals are usually nursed by sisters of mercy, and so are various other hospitals. Nurses belonging to associations, whether the order be a religious or a lay one, are forbidden to receive fees or gratuities from patients, whether rich or poor, all payments being made direct to the several societies. Recreation and rest for the busy workers are provided in the glorious Black Forest, where stands a beautiful home for nurses needing holidays.

### Everybody's Opinion.

*[Correspondence on all subjects is invited, but we cannot in any way be responsible for the opinions expressed by our correspondents. No communications can be entertained if the name and address of the correspondent is not given, or unless one side of the paper only be written on.]*

### NURSES AND THEIR FEES.

"Ex-Nurse" writes: In reply to "A Patient," who complains of the difficulty felt in obtaining skilled nursing for middle-class families, who are not really able to pay a trained nurse's fee, I think that if more nurses' co-operations, similar to the one in London, now working so successfully, were established, both docters and the general public would soon support them in preference to nursing institutions. But each nurse who became a member would have to agree to devote part of each year to nursing in families, where she would receive a fee in proportion to the family's income. The co-operations might also exempt nurses from paying percentage on such cases. Such an arrangement would at least partly meet the want felt, but I think it would be very unreasonable to expect nurses to reduce their fees to those who can really afford to pay; nurses' pay is hardly won money, and a nurse's life is short-lived, unless she can afford to rest between her cases, which she cannot do unless she is well paid. I know a nurse, working on her own account, who reduced her fee not long ago to a middle-class family, and worked for some months upon five hours' sleep out of the twenty-four, and very little recreation. She had to give up in consequence of her strength giving way; fortunately, she had a good home to go to, but had to rest for some weeks after it. Starting co-operations means outlay of money and consequently some risk, but there would be no fear of their ultimate success, if every one concerned would do what they could to help them on. Nurses' earnings by their means would go into their own pockets, as they ought to, instead of the middle-men's. Nurses would then also be able to save something for a rainy day and old age, which I scarcely think many can do with the salaries that are given nowadays.

### WANTED, A HOME FOR THE CURE OF HYSTERIA.

Can somebody help this correspondent, "St. Helena," who writes: Could you or any of your readers tell me of any place where it would be advisable to send a girl of 20 or so who suffers from hysteria? She is now able to get about and do many things, and is a most accomplished and amiable girl. She at one time brought herself to the last stage of prostration and starvation by steadfastly and cunningly avoiding all food, so that her life was despaired of by more than one eminent London physician. She still suffers from serious disabilities, and shows at times an aversion to food. It is considered advisable for her to be away from home, but her parents cannot afford for any length of time the three guineas which is the least charge they know of. But is there any suitable place where the charge is less?

### Medico-Psychological Association.

### CERTIFICATES OF PROFICIENCY IN NURSING.

The following candidates were successful at the examination for the certificate of proficiency in nursing, held in November, 1892.

Winson Green Asylum, Birmingham.—Male nurse: Alfred Yarnal. Female nurse: Bertha Holden.

Derby Borough Asylum.—Female nurse: Helen N. MacDonald.

Holloway Sanatorium.—Male nurses: William Aries, Alfred Gouriet, Edward John Green, Lambert Jenkins, Henry Ponsford, John Webber. Female nurses; Lydia Barrett, Clara Cowling, Maud D'Arcy, Elizabeth Annie Greatbatch, Kathleen Gleeve, Elizabeth Heraper, Annie Hughes, Minnie Julius, Emma Mary Pakenham.

Crichton Royal Asylum, Dumfries.—Female nurse: Jessie McLeod.

Kirklands Asylum, Bothwell.—Male nurse: John Macaskill.

Sunnyside Asylum, Montrose.—Male nurses: John Dunbar, Robert Emslie, John S. Massie. Female nurses: Annie Duncan, Christina Duncan, Elizabeth Findlay, Elizabeth McIntosh, Margaret Middleton, Mary McCall.

The next examination for this certificate will take place on Monday, the first day of May, 1893. Candidates can obtain from the registrar a schedule, which should be filled up and signed as required, and returned to him at least four weeks before the date of the examination. Letters of enquiry respecting this certificate should be addressed to Dr. Spence, Burntwood Asylum, near Lichfield, Staffordshire.

### Wants and Workers.

*Dipsomania.*—Will any reader kindly inform me of a home where a young man, addicted to drink, could be received on payment of moderate fee?—*Nurse.*

*The Melicent Home.*—Miss Tomlin, Rosslyn, Ryde, Isle of Wight, writes to us as follows: "Will any kind friends give some assistance to the Melicent Home at Sandown, Isle of Wight, for crippled children suffering from spine or hip disease, paralysis, or chest disorders, who have been discharged from hospital, but who require further care and nursing, and for whom sea air is essential? Help is greatly needed for the funds of the Home, and to supply the many wants of the little sufferers; gifts of blankets, good knitted stockings, and warm clothing, old or new, would be most acceptable. Children of suitable ages, when strong enough, are taught housework, and when ready for service are found situations; but children of all ages are admitted. The highest rate of payment is 8s. for a bad surgical case, but the matron will be delighted to forward every particular. I am well acquainted with this institution."

*A Home Needed.*—Will any one of affluence help build a small Home for Nurses in a poor neighbourhood, estimated at £500? The district nurses have been working since Jubilee year, but have no permanent Home. One lady has offered help provided others can be found. All particulars from "District Home" at this office with kind permission.

### Novelties for Nurses.

A RECENT visit to Messrs. Garrould's establishment in Edgware Road has introduced to our notice two very pretty new caps made by this firm. They have been specially designed to reduce the labour of the "making up" of a clean cap by a nurse to a merely nominal undertaking—for they are so constructed as to need no elaborate preparation for going to the laundry or for returning to immediate use. One shape is made of fine cambric, and trimmed with a goffered frill of the same, which is edged with very narrow Valenciennes lace. A running string regulates the shape and the size, and the adjustment of this is the only treatment required for converting an oblong piece of material into a very dainty cap, with strings to tie under the chin. It is moderate in price, and ought to wear well and be popular. The other novelty is made of fine muslin trimmed with rather wide lace, and the effect is very good, a cunningly-devised pleat being all the shaping required to make the cap ready for use. Only a fully-employed private nurse is, perhaps, perfectly capable of appreciating the advantage held out by these methods for economising her time. The cloak which is known as the "St. Angelus" is specially suited for winter weather and muddy days, as the large slit through which the arm passes also gives opportunity for the wearer to hold up her dress in an easy and satisfactory fashion whenever she wishes to do so. The pocket is contrived with much judgment in the most convenient position possible ; in this respect it is a contrast to many others at the present day, which seem specially designed to confuse and puzzle the fair wearers by their mysterious location. The "St. Angelus" is made of thick Melton cloth, thoroughly shrunk ; and so is the "St. Winifred," which has a full-gathered back and a wide fold in front to protect the wearer's arms. Both cloaks are of moderate prices.

### Appointments.

[It is requested that successful candidates will send a copy of their applications and testimonials, with date of election, to THE EDITOR, The Lodge, Porchester Square, W.]

GENERAL HOSPITAL, BRISTOL.—Nurse Alice Hill, who, as "Sister Riddle," has been connected with this hospital sixteen years, and has won the esteem of both surgeons and nurses, has been elected Night Superintendent here.

ST. SAVIOUR'S INFIRMARY, DULWICH.—Nurse Louisa Underhill has been appointed Sister to one of the wards of St. Saviour's Infirmary, Dulwich. Nurse Underhill has taken her three years' certificate of training at the Dreadnought Hospital, Greenwich.

BRIGHOUSE JOINT HOSPITAL.—Nurse Emily Reid has been appointed Matron to this hospital, where she has been acting as temporary Matron for six months. Nurse Reid was trained by the Bradford Nurse's Institute in medical, surgical, and fever-nursing.

SINGAPORE.—Nurse Laura M. Potter, head nurse of the Birmingham Borough Asylum at Rubery Hill, Bromsgrove, and formerly of the West Riding Asylum, Wakefield, has been appointed Matron of the Government Lunatic Asylum at Singapore.

GENERAL HOSPITAL, BRISTOL.—Nurse Sophie Morris was elected Assistant Matron at this hospital on November 30th. She received her three years' training at the General Hospital, Birmingham, and after this period she was placed in charge of the female operation wards, and afterwards became Sister-in-charge of the Operating Theatre. After nine years' work at the Birmingham General Hospital Nurse Morris was elected Night Superintendent at the Bristol Royal Infirmary, which post she held for fifteen months, and up to the date of her present appointment she has been acting in the same capacity at the General Hospital, Bristol. Nurse Morris holds admirable testimonials from doctors and matrons alike, and is in every way fitted for the work she has undertaken.

### For Reading to the Sick.

MELANCHOLY.

THE weariness of life which besets us in illness is often accompanied by deep melancholy, and our aim should be to fight against it with all our might. Some persons give way to despondency, even going half-way to meet their troubles, and dwelling on their weakness, their pains, their privations of all sorts, till, alas ! in the end doubting the justice of God, who has allowed trials to come upon them. In those days of clouds and darkness we say, Why should others be cheerful and we sad ? Our pains, our sufferings must be the greatest in the world ; they are far greater than any we ever heard of; and we become sullen and ill-tempered, and toss and moan in sheer impatience and desperation.

Mr. Keble gave excellent advice in his "Letters of Spiritual Counsel" by saying, "When you feel yourself overpowered by melancholy, the best way is to go out and do something kind for, or to, somebody or other" ; and Mr. F. Parnell, in treating of a kindred subject, observes, "When I dig a man out of trouble, the hole he leaves behind him is the grave in which I bury my own troubles," so that when we feel our dull moods coming on, let us quickly and with determination turn to the first work we can at the moment do—for we can always find something, if not great works, then little ones. We are not now speaking to, or of those who are passing through a crisis of fever or are at the point of death ; such sufferers can neither work nor think while in those straits ; but we would point out how people can act who linger for years with one of those mysterious complaints in which the patient simply exists, for it is hardly living, and certainly is not dying. Yet it is in the wards of an incurable hospital that one sees the greatest proportion of cheerful faces. Many a one among them has doubtless passed through the "Slough of Despond," and, like "Christian" in the "Pilgrim's Progress," come out safely through the grace of God, and can now work on in His strength. It is a touching sight to see the poor weak hands, which ordinarily would be a sufficient excuse for idleness, busying themselves in needlework, or with one poor crippled member making warm garments in crochet or knitting, which prove "comforters" in deed as well as in name to both giver and receiver. If work, then, for others, whether in health or sickness, be the panacea, the universal medicine for the cure of melancholy, let us take care that we do it in the name of the Lord, or the effects will not be lasting. It is He who strengthens the weak arm and the feeble knees. He shows us the Son of God hanging, on Calvary for the love of man—can we turn away untouched from the sight ? The Saviour who worked and suffered all His life for the souls of men is waiting now, watching for a change to come over our sullen minds, longing for that turn of will which may let in again the glad tide of light, and joy, and health to the dull and silent heart.

### Some Australian Experiences.

(*Continued from page* lxxxviii.)

### OFF TO THE GOLD FIELDS.

After my return to Brisbane I had an offer to go up to one of the new North Queensland gold-fields. Being anxious to see all I could, I accepted the post of matron, at £100 per annum, thinking I might also save a little in the country. You shall see how I did it.

It was a bit of a wrench leaving Brisbane—I had so many kind friends—and this place was over a fortnight's journey, all among strangers too; still there was the novelty of seeing life on a gold-field. As I heard it was a nice hospital I felt little consoled; and private work was not too plentiful in Brisbane just then.

Having been trained amongst miners, I thought I should feel at home. I had to learn the difference between coal miners and gold miners. The former are rough, but kinder hearts do not beat anywhere. The latter—— Well, you shall judge later on.

The distances in Australia between different parts are so great that although my new appointment was in Queensland, I had to travel a fortnight before reaching my destination, which lay beyond the extreme northern point of the colony, and almost the last piece of civilisation on the brink of a trackless country as yet unexplored.

I took my place in the very comfortable steamer *Quiraing*, which, although not one of the grandest, is certainly a most pleasant boat to travel in, one distinct advantage in it is taking this tropical voyage is the deck cabins, which are so cool. I was placed in the captain's charge, being the only lady going the whole length of the journey, and such a journey.

I thought I had seen most of the beauties of the coast and favourite spots of both Melbourne, Sydney, and Brisbane, which are supposed to comprise Australian scenery, but all I had ever seen faded before the glories of the Torres Straits, inside the great barrier reef of Australia.

We called at several ports up to Townsville, where the best part of the scenery begins. It is perfectly bewildering. Our captain, who is both a naturalist and an antiquarian, told us all the incidents connected with the different islands, both interesting and touching in character; the poor people who lost their lives through the "blacks" are so numerous.

We then went on to Thursday Island, one of such a pretty group. Here we again landed, and the Governor (the Hon. John Douglas) most kindly entertained us at tea at the Residency—such a pretty house, commanding a view of the principal islands, which can be plainly seen through the telescope on the verandah.

This is the centre of the great pearl shell fishery, which employs a large coloured population. After spending a very pleasant evening we returned to our boat, and that night left all the beauties behind, for the morning found us in the great Gulf of Carpentaria, where lay our destination, Normanton and Burketown being the last ports on that side of the coast. The large steamers do not go up the rivers, as a rule, in the north, but are met by "tugs." We, therefore, had to bid good-bye to our genial captain and board a small "tug" to convey us to Normanton, which generally takes from 12 to 18 hours. What a terrible trip it was, the heat intense, sandflies, mosquitos, and the smell of decayed vegetation on the river banks, not to mention the odours from the engines, kitchen, &c., &c. We went on board at night; a very nice, kind priest looked after me, and we landed at about ten in the morning.

The first appearance of Normanton was a decided shock to me. I had never seen a place so completely devoid of trees and vegetation, and so flat—it seemed nothing but dust and glare, and such heat, only fit for "black-fellows" was my mental obsevation.

There were buggies waiting to take us to our hotel, where we had to stay a day and night before the coach started for the Croydon gold-field, distance 125 miles.

"Heley's Hotel," Normanton, is certainly a marvel of comfort in the wilderness, and very welcome to the tired traveller. We found breakfast waiting, after which the first thing to be done was get rid of all superfluous clothing.

More suitably arranged I sallied forth to inspect the town. The new railway was just begun, so we were to travel twenty-five miles by that to meet the coach. Such a funny rough train; the carriges were like horse-boxes, only not quite so comfortable. It took us three hours to get over that twenty-five miles, and we were deposited at a real "bush shanty," where breakfast was supposed to be in readiness. I never had such a meal—horribly tough salt beef, fried in pasty sort of butter, all nasty and soddened with fat, and smoked tea without sugar.

Here I was met by the nurse who had left Croydon, my predecessor, who met me with tears in her eyes, saying, "Oh! Sister, I am sorry it is you; don't stay up there, it is a frightful place." This was not encouraging. We had a chat over our smoky tea, then bid a hasty farewell—she going to my steamer, I to my new field of action. Here I saw the first "bush stage coach," which carries "Her Majesty's Mail." Such a coach! I thought of the "White Horse" Cellars, and wondered how this would look in Hyde Park. Well, one thing, we had fine horses. Cobb and Co. feed their animals well, so there was no fear of them failing us. By eight a.m. we had started, I sat on the box. The heat was something awful. There had been no rain for two years, so all the creeks were dry, and the waterholes we came upon now and then were nothing but nasty, thick water. Yet we drank it. I had ulcerated lips for a fortnight in consequence. At twelve a.m. we stopped at a "stage" to change horses and have dinner—boiled salt beef, tea, and bread, for which we paid 2s. 6d. The same had been charged for breakfast.

Then off again through the bush—nothing but flat bush—bumping, thumping, over logs and ant hills, and when we came to the dry creeks, we swooped down the banks and up the other side in a most marvellous manner. Sometimes the coach does go over, and no wonder, it is a work of art to keep your seat.

All this time the sun is pouring down in a merciless manner. The trees are so thin they afford very little protection, and a sunshade is out of the question with three on the box. At four p.m. we again alighted, and were delighted to find tea with goat's milk—salt beef, of course, nothing else will keep in the heat. Here also we got a little water to wash in, but oh! such filthy stuff. Yet we were thankful for it, and felt better.

By this time we had got through the worst of the heat, and off we went again. Same thing—flat bush, logs, and creeks; it was wearisome. Of course, in the tropics there is no twilight. As soon as the sun went down we were in utter darkness, for the coachman had forgotten his candles, and no one had any to spare at the "stages." I shall never forget that drive in the dark through the "bush," which lasted five hours and a half; it was awful. The coachman must have been a splendid driver to have got us through. I never held on to anything like I did to the back of the seat, and, I must add, to the gentleman next me. He was most kind to me on the way, and amused me by telling me of the different celebrities of the place. Not least among them was a very small man called "Terrible Billy," who gave the police a lively time. I saw him afterwards in the jail.

(*To be continued.*)

## Figures and Tables

**Figure f1:**